# Remarks, Gleanings, and Extracts

**Published:** 1874-11

**Authors:** Swan M. Burnett

**Affiliations:** Knoxville, Tennessee


### REMARKS, GLEANINGS AND EXTRACTS.

BY SWAN M. BURNETT, M.D., OF KNOXVILLE, TENNESSEE.

*Physiological Action of Hydrocyanic Acid.*—In the London *Practitioner* for September is a translation of an article by Dr. Baehm, detailing the experiments of himself and Dr. Knie, made at the Physiological Institute, at Dorpot, relative to the action of hydrocyanic acid on the system, and especially in regard to the antidotal action of atropine, which has been contended for by Dr. Preyer. The following is a resumé of the paper:

1. The operation of prussic acid is directed upon the central nervous system, whose functions are annihilated by large doses, after a brief excitement or increase.

2. The lesions of respiration and circulation arise from analogous changes in the activity of their centres in the medulla oblongata.

3. The vagus plays no part, either in the effect of prussic acid on respiration, or in its effects on the heart.

4. Atropine is not an antidote to prussic acid. The only rational treatment of this poisoning is the persevering performance of artificial respiration.

*Hospital Observations Upon Negro Soldiers.*—Dr. McDowell, of New Jersey, gives, in the Sept. No. of the *American Practitioner*, the results of his observations upon the diseases and pathology of the negro as observed in army hospital practice.

Though the chest measurements of negro recruits were large, he found these same individuals suffer severely with pneumonia, and lie rapidly. Post mortem examination showed the cause of this occurrence. Though the chest measured well, the lungs were small, the largely developed mammæ and pectoral muscles giving the fullness to the chest.

The brain he found to be smaller, and to increase in size *pari pass* with the increase of admixture of Caucasian blood.

The negro liver was found to be larger than that of the white man, while the lower bowels were smaller. He refers the constipation from which the negro soldiers suffered to this latter circumstance. Ague cake as a sequella to malarial fever he never found among the negroes.

The prognosis of disease among this class of soldiery he found much more grave than among the whites, and the stimulating plan of treatment was necessary almost from the beginning.

Cerebro-spinal meningitis was a frequent and very fatal disease among them.

*Antidotes to Poisoning by Strychnine.*—Professor Ranieri Bellini, after conducting a long series of experiments on poisoning by strychnia and its salts, arrives at the opinion that the best antidotes are tannic acid and tannin, chlorine, and the tincture of iodine and bromine. Chlorine, he maintains, attacks the strychnia even when it is diffused through the system; for he found that in rabbits poisoned with the sulphate of the alkaloid, on being made to inhale chlorine gas in such quantity as was not sufficient in itself to kill, the convulsions were retarded, and were milder when they occurred; death was also less rapid. The author further observed, that when strychnia was exhibited with pyrogallic acid, the convulsions were retarded for the space of half an hour, in comparison with other experiments in which the alkaloid was given by itself. Professor Bellini believes that this arrest in symptoms is not dependent on the acid acting chemically on the strychnia, but only through the astringent effects produced by the acid on the mucous membrane of the stomach, whereby the absorption of the poison is rendered difficult. The same author, dwelling on the frog-test for strychnia, asserts that this test is not to be trusted, inasmuch as other poisons produce the tetanic symptoms, although in a less degree. He adds, in speaking of the effects of the antidotes to which reference has been made, that he trusts his results will have a bearing not only on the treatment of strychnia tetanus, but on traumatic and idiopathic tetanic disease.

*A Preparation for Treating Diarrhœa of Nursing Infants.*—Bismuth, with Pepsin; best French brandy; Ext. geranium fluid; gum arabic water. Use bismuth merely to hold it in suspension.

*Treatment of Inflamed Hemorrhoids in Parturient Women.*—A number of remedies have been advised for this condition, which presents a certain gravity, especially in nervous women. Most of these are, unfortunately, inefficacious. Leeches to the tumors, or their incision, are not without danger, and only afford a transient relief. Ointments, liniments, suppositories, and the like, produce but little effect on the inflamed vessels, and yet the patient must be relieved from her insupportable pains. A small piece of ice is to be placed in a little bag of rubber or gold-beater's skin, and applied to the tumor. The ice is to be replaced as fast as it melts. It is very rarely that at the end of an hour or two the pain is not very much diminished. The treatment may be continued for a part of the day and repeated the next, but care should be taken to envelop the bag with fine moist linen, so as to render the application less direct. It is also necessary, when about to discontinue the refrigeration, to let the ice melt completely, and permit the water to attain the temperature of the bed, to prevent a reaction which might reinduce pain.—*Gazette de Joulin* and *La France Medicale*, September, 1874.

*Neuralgia in Infants.*—Children from two to six weeks old, especially males, suffer frequently with attacks of pain in the bowels, coming on about midnight, and lasting until four or five in the morning. This enteralgia does not seem to be caused by any fæcal accumulations; it is very noticeable, however, that during the paroxysm they pass no water, and at the end of it a large quantity of pale-colored urine comes away, as after an hysterical attack. The cause of this retention of urine is unknown. The disease affects children of all classes of society indiscriminately, without reference to their hygienic condition.

The remedy recommended by Dr. Boyd (*Edinburgh Medical Journal*, February, 1873; Schmidt's Jahrbucher, 1873, No. 2), is spiritus ætheris nitrosi, eight or ten drops in a drachm of water.

Immediately afterwards, with escape of wind and the passage of a considerable quantity of urine, the crying ceases, and the little patient goes to sleep.—*Boston Medical and Surgical Journal.*

*Local Action of Ipecacuanha.*—Dr. De Mussy, of Paris, in the London *Practitioner* for September, gives some observations on the local action of ipecacuanha. In dysentery (for which it has long been given *per orem*) he uses a decoction (ʒj. of the root to ℥v. of boiling water) as an enema. But strangest of all he has used a decoction of half this strength as a topical application to granulated lids accompanied with ulcer of the cornea. Dr. Galezowski, to whom he related the case, also tried it in several cases of *subacute* inflammation, and with some success.

*Resuscitation from Chloroform-Narcosis* (*The New Orleans Medical and Surgical Journal*, November, 1873).—In the course of an extended experience in the administration of chloroform, it has happened three times to Dr. M. Schuppert, to all appearances, the narcotized subject died,—that is, respiration ceased, the heart stopped beating, and muscular contractibility became extinct. The method he adopted for resuscitating these patients consisted in reversing the body, either by hanging them up by the feet or laying them over a bed or table, so that the greater part of the body with the head hung down. In that position artificial respiration was also tried. In one case five minutes elapsed before there was a natural inhalation. All of them recovered. Dr. Schuppert believes that in cases of death from chloroform the primary cause of the cessation of the respiration and circulation rests in anæmia of the brain, and not in impregnation of the blood with carbonic acid. —*Phil. Med. Times.*

*Phosphorus Pill.*—Take of Phosphorus, 2 grains; Balsam of Tolu, 120 grains; Yellow Wax, 60 grains.

Put the phosphorus and balsam of tolu into a Wedgwood mortar about half-full of hot water, and when the phosphorus has melted and the balsam has become sufficiently soft, rub them together beneath the surface of the water until no particles of phosphorus are visible, the temperature of the water being maintained at or near to 140°. Add now the wax, and as it softens mix it thoroughly with the other ingredients. Allow the mass to cool without being exposed to the air, and keep it in a bottle immersed in cold water. It may be softened with a few drops of rectified spirit when made into pills.

Dose—Three to six grains.

*Colorless Tincture of Iodine.*—℞. Tincture of iodine, pure glycerine, aa. ℥j.; sulphite of soda, ʒj. Rub the salt to a powder in a small mortar, and add the glycerine gradually; then pour in the tincture of iodine, and triturate gently until a solution is effected and the mixture assumes an amber color. It is asserted that the properties of iodine are increased by the addition of the sulphate of soda, and that the glycerine enhances the value and convenience of the preparation for local application.—*Cincinnati Lancet and Observer*, January, 1874.

*Purgative Mixture of Vienna.*—The *Journal de Pharmacie et de Chimie* gives the following:

Manna, 66 grammes; senna, 10 grammes; cream of tarter, 4; coriander raisins, aa 2 grammes; water, 320 grammes. Boil until the water is reduced to 190 grammes. Take before breakfast.

*Danger of Intra-Uterine Injections.*—The *Gazette de Joulin* gives the details of two cases, which show that while intra-uterine injections are energetic agents in modifying the conditions of this mucous cavity, they should be employed with caution.

In one case, though the patient had become enfeebled by repeated hemorrhage, she endured, without suffering inconvenience, two injections of the uterine cavity. A third, consisting of a weak infusion of chamomile and diluted perchloride of iron, was succeeded by death in thirty hours after decided symptoms of subacute peritonitis. The mucous lining of the uterus and right fallopian tube, and the adjacent peritoneal surface, were found, after death, covered with an ink-black clot and presenting unmistakable evidences of inflammation.—*Medical Examiner.*

*Chloride of Potassium in Epilepsy.*—Dr. Lander gives (*Scalpel*, Belgium) chloride of potassium instead of bromide of potassium in epilepsy. He mentions the following advantages in the employment of the substance: It is more active, is but one-sixth of the cost, and has not the secondary effects of the bromide. He begins with small doses, but has been able to continue the use of the substance for months without any inconvenience, in daily doses of from one drachm to a drachm and a halt. According to Dr. Lander, bromide of potassium is transformed into the chloride in the stomach. This is, therefore, an additional reason for prescribing it at once in this latter form.—*Lancet.*

*Charcoal and Tar as a Surgical Dressing.*—The London *Lancet* strongly recommends the use of a mixture of charcoal and coal tar, containing thirty-three per cent. of the latter, in pulverized form, as a dressing for wounds. The powder exercises no irritative action, and is easily removed by lotions of cold water. The charcoal absorbs gases due to fermentation, coagulates the albumen, and prevents decomposition, in this respect materially aiding the action of the carbolic acid contained in the coal tar. For wounds which cannot bear the contact of the powder, 100 parts of pulverized coal tar are macerated for some hours in 400 parts of rather weak alcohol. The solution is said to be very efficacious.

*Treatment of Zona by Collodeon and Morphia.*—Dr. Bourdon, of Hospital la Charitè, after having tried a great many local means for treating the above disease, and checking the intense pain, has definitely adopted the following plan: Without opening the vesicles, he paints all the diseased surface with a combination of collodeon and morphia—collodeon, one ounce; morphia, eight grains. The mixture must be put on pretty thickly.—*Lancet.*—*American Practitioner.*

*Nutritious Injections—a New Method of Nourishing Patients by the Anus.*—M. Leube has had the idea of rendering the digestion in the large intestine very active by carrying into this organ, simultaneous, digestible substances and a substance which is digestive. The pancreas of the hog constitutes the latter. The mass to be digested is made in the following manner: Fifty to one hundred grammes of the pancreas of the hog or ox, carefully deprived of its fatty tissue, are chopped fine and mixed with one hundred and fifty to two hundred grammes of beef. The two substances are pounded in a mortar with warm water, a magma being formed which is injected by means of a syringe, the nozzle of which is of large calibre. The injections thus composed have given good results with dogs. A fecal mass is formed after the injections, which is quite analogous to the ordinary matters; the fat and the albumen are digested by the large intestine. This method of nutrition has been applied by the author to two patients. In one case there was a cancer of the upper portion of the digestive tube; in the other the patient could not receive any aliment without rejecting it by vomiting. In these cases the pancreatic substances did not cause any diarrhœa; they remained in the intestine for from twelve to thirty-six hours without producing any evacuations, and the patients did not experience any pain. After the injections the stools became more full; but at first the injections were not entirely retained, the patients rejecting a part of the injected mass undigested. This *melange* is, according to the author, superior to all the substances which are recommended for nourishing by the large intestines.—*Gazette Hebdomadaire* and *La Tribune Medicale. No.* 266, 1874—*New York Medical Journal.*

*Acute Ergot-Poisoning.*—Dr. Henschel reports in the *New York Medical Record* a case where thirty minims of Squibb's Fluid Extract of Ergot were given by mistake to an infant. Soon after there was severe abdominal pains occurring every fifteen minutes and lasting hardly sixty seconds. There were slight tetanic contractions of the muscles of the face and extremities. Diarrhœa set in four hours after administration and continued fourteen days. He believes that this case proves that it is the uterine contractions, and not the ergot that destroys the child during labor.

*Iodide of Potassium in Acute and Chronic Bronchitis and Asthma.* Dr. Spurgin, of the York Dispensary, (*British Med. Jour.*) has been using iodide of potassium with much success in these complaints. He generally gives it in connection with carbonate of ammonia; sometimes adding tinct. belladonna and ipeccacuanha wine.—*Medical News and Library.*

*Is Labor Protracted by Early Spontaneous Rupture of the Membranes.*—By G. W. H. Kemper, M.D.—The above question is, I believe, answered affirmatively by all our text-books, and the student goes from his books to the bedside impressed with this idea. The most recent obstetric authority, Leishman, thus unqualifiedly expresses himself as to the effect of early rupture of the membranes (Obstetrics, p. 255): "And the result when that occurs is, as every one knows, protracted labor and increased risk to the child." I well remember my own anxiety attending my first case of "dry labor." A larger experience and careful observation have led me to believe that the statement we have quoted is not to be received as absolute truth. This belief has been confirmed by reading the following extract from the Obstetrical Journal of Great Britain and Ireland, December, 1873, p. 629: "Having described the functions of the bag of waters, Dr. Gartipny proves by the notes of two thousand deliveries, that its spontaneous rupture is of frequent occurrence. The premature flow of the waters hastens the labor, and exercises no injurious influence on the mother or child. Its occurrence is therefore favorable when pregnancy has arrived at its full term.—*American Practitioner.*

*Chlorodyne.*—Dr. McGeachy, (says *Canda Lancet*) of Iona, sends us the following formula for chlorodyne. He has used it for the past eight or nine months, and finds it as good as Collis Browne's or any in the market: ℞. Morph. sulph., grs. iv.; chloroform, eth. sulphric, aa ʒj; acid hydrocyanic (S.) ♏xxx.; ext. cannab. indic., grs. viij.; ol. menth. pip., ♏viij.; tinct. capsici, ʒss.; acid phosphoric, dil., ℥ss.; golden syrup, ad., ℥j. Mix in a two-ounce bottle the hemp, chloroform, ether, capsicum, and morphine previously dissolved in the acid, and shake until complete solution takes place. If necessary, strain rapidly through muslin wet in alcohol. Add the remaining ingredients, and preserve in a dark bottle.

*Croton Chloral Hydrate in Intolerance of Light.*—Dr. Bader, of Guy's, communicates in the *Med. Times and Gazette* ten cases of intolerance of light, dependent upon corneal and iritic inflammation, in which benefit was secured by the administration of croton chloral hydrate. He gives it in doses of from five to ten grains, three or four times a day.

*Chloral in Premature Labor.*—M. Martineau makes use of enemata containing sixteen grains chloral hydrate in four ounces of water, in cases where labor has set in. If the first injection does not quiet the pains, it may be repeated at intervals of several hours. Chloroform is said to be formed in the bowel by the action of the fæces, which are usually alkaline.

*Notes of Hospital Practice, Charity Hospital, New York.—Mucous patches* yield in a few days to the internal administration of one-fourth grain of green iodide of mercury, together with the local application of the solution of acid nitrate of mercury. The same caustic proves very serviceable in condylomata, and is used in preference to others.

BUBOES.—If these are the results of cancroids, any attempt to prevent their suppuration by pressure, with application of tinct. iodide, usually fails. When suppuration does take place, the dissection out of the gland by means of the handle of the scalpel is the best method to promote a speedy cure. The cavity is filled with balsam of Peru and oakum, in order to get the sore to heal from the bottom.

EPIDIDYMITIS.—Many varieties of treatment have been had recourse to, but the one that gives the best satisfaction is the tobacco poultice. The method used is to make a poultice of tobacco leaves, and apply it to the scrotum at night. In the morning it is found that much of the pain has disappeared. The poultice serves a double purpose—first, that of an ordinary poultice; and secondly, that of a depressant and nauseant—*New York Med. Jour—Clinic.*

[For the past several years we have used scarcely any other treatment for epididymitis than the tobacco poultice, and find it acts most admirably.—B.]

We have received from Dr. B. Joy Jeffries, of Boston, a report of 105 cases of operation for cataract, performed by him in private and hospital practice. All the extractions (with a few exceptions) were done by Graefe's method, which he now esteems superior to the old flap method. A condensed history of the cases and results of the operation are given, which will be of value to the statistician of cataract operations.

*Treatment of Burns and Scalds.*—Dr. de Breyne highly recommends the following treatment in *L'Union Pharmaceutiqüe*: Hydrate of lime (newly precipitated), forty-five grains; glycerine, five ounces; chloric ether, forty-five drops. It makes up a transparent, colorless liquid, with an agreeable odor, and an alkaline reaction, according to the dose of hydrate of lime. It calms the pain, and prevents or abates inflammation.

*Cannibal's Tastes.*—It is said Cannibals do not like the flesh of the white man so well as that of the negro. They find white flesh bitter and salt, but still is not so easily preserved as that of the negro, which drys well.

Rokitansky, it appears, will still continue to hold his position as professor of pathological anatomy at Vienna.

